# Prognostic Significance of Triglyceride-Glucose Index for Adverse Cardiovascular Events in Patients With Coronary Artery Disease: A Systematic Review and Meta-Analysis

**DOI:** 10.3389/fcvm.2021.774781

**Published:** 2021-12-02

**Authors:** Jin-Wen Luo, Wen-Hui Duan, Yan-Qiao Yu, Lei Song, Da-Zhuo Shi

**Affiliations:** ^1^National Clinical Research Center for Chinese Medicine Cardiology, Xiyuan Hospital, China Academy of Chinese Medical Science, Beijing, China; ^2^Graduate School, Beijing University of Chinese Medicine, Beijing, China

**Keywords:** triglyceride-glucose index, insulin resistance, coronary artery disease, adverse cardiovascular events, meta-analysis

## Abstract

**Background:** Insulin resistance (IR) represents a critical regulator in the development and progress of coronary artery disease (CAD). Triglyceride-glucose (TyG) index, a novel surrogate biomarker of IR, has been implicated in several cardiovascular diseases. Accordingly, we conduct a meta-analysis to elucidate the relationship between TyG index and adverse cardiovascular events in patients with CAD.

**Methods:** To identify the studies examining the predictive capacity of the TyG index for adverse cardiovascular events in the setting of CAD, we performed a comprehensive literature retrieval of Scopus, PubMed, EMBASE, and Web of Science, from the inception of databases to October 5, 2021. We pooled the adjusted hazard ratio (HR) along with 95% CI using a random-effects model. The primary outcome was a composite of major adverse cardiovascular events (MACEs), including all-cause death, cardiovascular death (CV death), myocardial infarction (MI), stroke, hospitalization for unstable angina or heart failure, and revascularization. The secondary outcomes were all-cause death, CV death, MI, stroke, and revascularization. Additionally, we conducted subgroup analyses stratified by diabetes status, age, body mass index (BMI), low-density lipoprotein cholesterol (LDL-C), category of TyG index, sample size, follow-up duration, and study design.

**Results:** About 12 studies involving 28,795 patients with CAD were finally taken into the quantitative analysis. Our findings showed that there was a 2.14-fold higher risk of MACEs among CAD populations in the highest TyG group compared with those in the lowest TyG group (HR: 2.14, 95% CI: 1.69–2.71, *P* < 0.001). A greater risk of MACEs was observed in participants with higher BMI than those with lower BMI (*P* = 0.03 for interaction). In the analysis of secondary outcomes, we also observed a markedly increased risk of MI, stroke, and revascularization in the highest TyG group compared with the lowest TyG group. No evidence of a significant association between TyG index and CV mortality or all-cause mortality in patients with CAD was identified.

**Conclusions:** The elevated TyG index is a promising predictive factor of adverse cardiovascular events in patients with CAD.

**Systematic Review Registration:** https://www.crd.york.ac.uk/PROSPERO, identifier: CRD42021228521.

## Introduction

Despite the guideline-directed therapy, coronary artery disease (CAD) is still the primary cause of death globally (1, 2). Although optimal treatments including drug therapy and revascularization effectively decrease the incidence of chest pain, individuals with CAD remain at an increased risk of recurrent adverse cardiovascular events (3). Therefore, it is crucial to identify the CAD population with a high risk of future cardiovascular events, which may contribute to the optimization of clinical management.

Insulin resistance (IR) is characterized by metabolism disorders, especially abnormal glucose, and lipid metabolism, leading to aggravation of atherosclerosis in the coronary artery (4, 5). Recently, a novel convenient biomarker of IR, Triglyceride-glucose (TyG) index calculated by the formula ln [fasting triglycerides (mg/dl) × fasting plasma glucose (mg/dl)/2], has elicited the interests of researchers because of its superior performance in the estimation of IR than a homeostatic model assessment of IR (HOMA-IR) (6–9). Certain studies in humans showed that TyG index was positively correlated with coronary artery calcification (10), coronary plaque progression (11), and subclinical myocardial injury (12), providing plausibility of the prognostic significance of TyG index in CAD. As a product of triglycerides and plasma glucose, the TyG index may provide additional information for the risk assessment of adverse cardiovascular events. Consistent clinical data have indicated that elevated TyG index was associated with a higher incidence of CAD (13–15). However, there are conflicting data regarding the predictive capacity of the TyG index for adverse events in patients with CAD (16–18). Thus, we conducted a meta-analysis to investigate the relationship between the TyG index and poor prognosis among patients with CAD.

## Methods

### Search Strategy and Eligibility Criteria

This meta-analysis has been registered in PROSPERO (https://www.crd.york.ac.uk/PROSPERO). The registration number is CRD42021228521. We performed this meta-analysis in accordance with the recommendations of the Meta-analysis of Observational Studies in Epidemiology group (19). In June 2021, to identify studies investigating the relationship of the TyG index with the risk of adverse outcomes in CAD, two reviewers independently conducted a systematic literature search of four databases (PubMed, Scopus, EMBASE, and Web of Science). On October 5, 2021, a repeat literature retrieval was conducted to supplement the latest research. Search terms included: “triglyceride-glucose index” OR “TyG index” AND “coronary artery disease” OR “coronary heart disease” OR “ischemic heart disease” OR “myocardial infarction” OR “stable angina” OR “unstable angina.” The literature retrieval was restricted to human studies published in English. A detailed search method of PubMed is presented in Supplementary File 1.

The inclusion criteria were: (1) observational studies (cohort study or nest case-control study); (2) participants were diagnosed with CAD (≥18 years old); (3) participants were exposed to different levels of TyG index at baseline; (4) the outcomes of interest were composite cardiovascular events, all-cause death, CV death, MI, stroke, and revascularization; (5) Adjusted hazard ratio (HR) with 95% CI from multivariate Cox regression models was available. The exclusion criteria were: (1) follow-up duration <3 months; (2) non-English works of literature; (3) duplicate reports, if studies including the same participants or overlapping participants were published, the study with the largest sample size was chosen.

### Data Extraction and Quality Evaluation

Two investigators independently abstracted the following data from each enrolled study: (1) basic characteristics of eligible study: name of the first author, publication year, country origin, sample size, and follow-up duration; (2) baseline demographic and clinical characteristics of subjects: age, sex, diabetes status, body mass index (BMI), low-density lipoprotein cholesterol (LDL-C) as well as the highest TyG index group, and lowest TyG index group; (3) outcomes: adjusted HRs for all-cause death, cardiovascular death (CV death), myocardial infarction (MI), stroke, revascularization, and a composite of cardiovascular events. In instances where there was insufficient information, we contacted the corresponding author. If disagreements occurred, an expert (WH Duan) in the field was consulted and made a judgment.

To assess the methodological quality of each selected study, we adopted the Newcastle-Ottawa-Scale (NOS) (20) designed for case-control study and cohort study. The NOS is mainly comprised of three dimensions: selection of participants, comparability among groups, as well as an outcome assessment. The NOS score ranges from 0 to 9 stars. Articles rated as 7 stars or above are of high quality; articles rated as 4 to 6 stars are of fair quality; articles rated as <4 stars are of low quality (21, 22).

### Statistical Analysis

Our primary outcome was major adverse cardiovascular events (MACEs), including all-cause death, CV death, MI, stroke, hospitalization for unstable angina or heart failure, and revascularization. The secondary outcomes were all-cause death, CV death, MI, stroke, and revascularization. To assess the relationship of the TyG index with the risk of future poor prognosis in individuals with CAD, we pooled the adjusted HRs and 95% CIs. If more than one model was used for the multivariate analysis, the most fully adjusted one was chosen. If outcomes were analyzed as relative risk (RR), we considered RR as an approximate HR in the meta-analysis (23). If the TyG index was evaluated as a categorical variable, the HR comparing the highest group of TyG index to the lowest group of TyG index was calculated. If the TyG index was evaluated as a continuous variable, the HR representing the risk of per 1-SD increment of the TyG index was calculated. *I*^2^ statistic and Cochran's *Q*-test were used as a measure of heterogeneity across the included studies (24). If there was significant heterogeneity (*I*^2^ > 50% or *P* < 0.05), sensitivity analysis, meta-regression analyses as well as subgroup analyses were conducted to explore the potential sources of heterogeneity. The random-effects model was used as the primary statistical analysis model in the estimation of pooled HR, even without significant heterogeneity, owing to the unavoidable clinical and methodological heterogeneity (e.g., demographics, medical history, medication, laboratory test of TyG index, and adjusted factors).

Sensitivity analysis by omitting one study at a time was conducted to assess the impact of every study on global HR. Meta-regression identifying the potential factors that might influence the pooled HR analyses were performed only if covariates were available in at least 10 studies. Moreover, we conducted subgroup analyses stratified by diabetes mellitus (DM) (with DM or without DM), age (<60 or ≥60), BMI (<26 or ≥26), LDL-C (<1.8 or ≥1.8), category of TyG index (median or tertiles), sample size (<2,000 or ≥2,000), follow-up duration (<36 or ≥36), study design (retrospective or prospective) and treatment (percutaneous coronary intervention, PCI). To assess the publication bias, we performed Begg's test or Egger's test (25, 26). *P* < 0.05 was considered as statistically significant. Statistical analyses were performed using STATA software version 12 (StataCorp LP, College Station, TX, USA).

## Results

### Characteristics of the Included Studies

**Figure 1:**
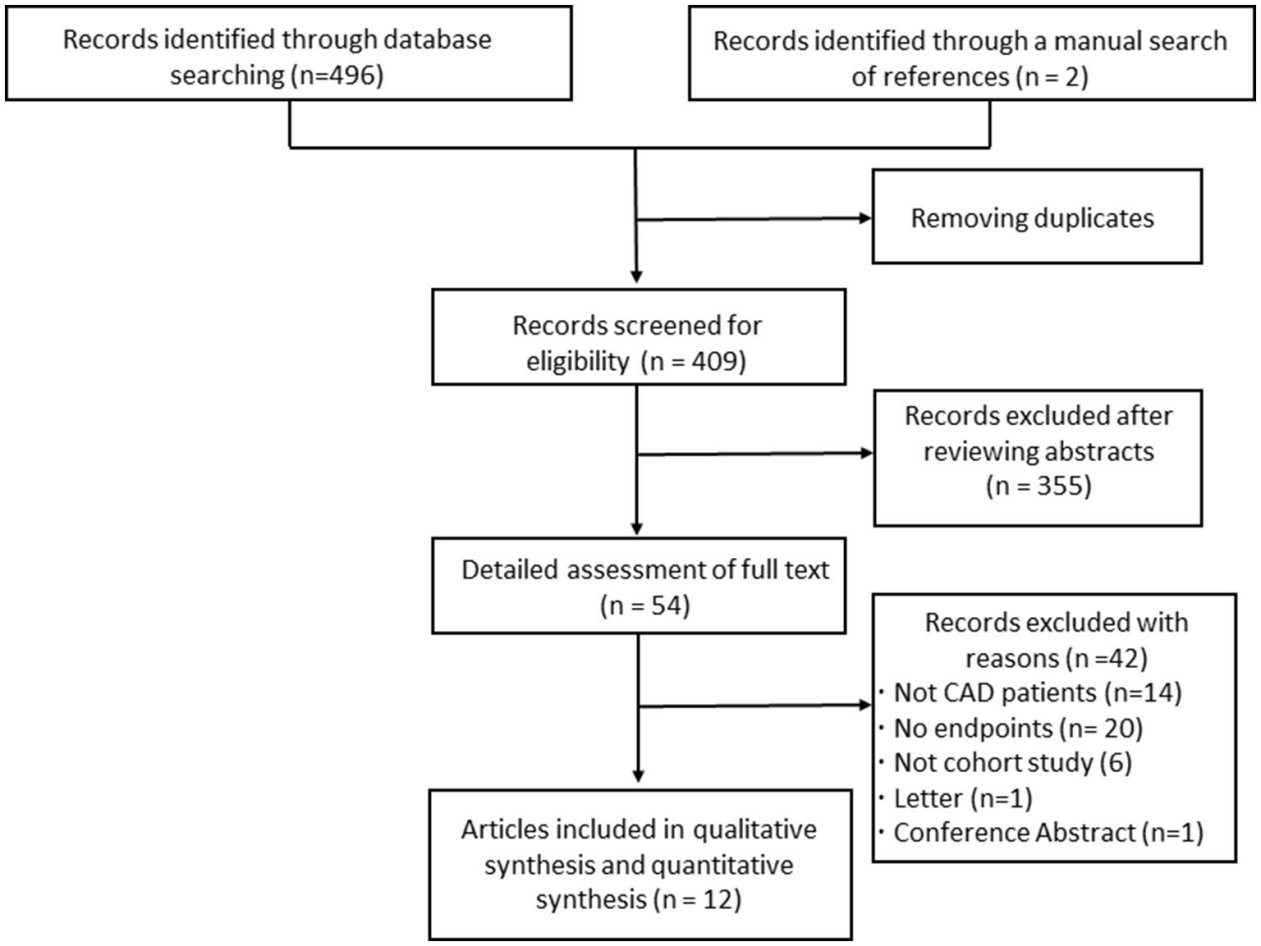
Flowchart of the study selection process and results.

Figure 1 shows the literature selection process. About 12 studies (27–38) with a total of 28,795 patients with CAD were finally included in the qualitative and quantitative analysis. All the included studies were published within the last 5 years and were situated in China. Of the 12 studies, one (27) enrolled stable CAD, 10 (28–35, 37, 38) enrolled acute coronary syndrome (ACS), and one (36) enrolled both chronic coronary syndrome and ACS. The average age of 12 studies ranged from 55.7 to 66.3 years. The proportion of men ranged from 55.9 to 79.4%. The follow-up duration ranged from 12 to 48 months. Four studies (30, 31, 33, 34) included diabetic patients; three studies (32, 35, 36) included non-diabetic patients; five (27–29, 37, 38) studies included both diabetic patients and non-diabetic patients. Methodological quality assessment using NOS indicated the good quality of all enrolled works of literature (Supplementary File 2). The main characteristics of 12 studies are shown in Table 1. The definition of MACEs and adjusted covariates in the individual enrolled studies are presented in Supplementary File 3.

**Table 1:** Characteristics of participants in the 12 included studies.

**Study**	**Country**	**Study design**	**Subjects**	**Sample size**	**Male (%)**	**Mean age (years)**	**Follow-Up (months)**	**Diabetes (%)**	**BMI (kg/m^**2**^)**	**The highest TyG index group vs. the lowest TyG index group**	**Mean LDL-C(mmol/L)**
Jin et al. (27)	China	Prospective nested case-control	Stable CAD	1,740	72.1	59.5	36	26.9	25.75	<8.40 vs. >9.17	2.51
Mao et al. (28)	China	Prospective cohort study	NSTE-ACS	438	67.4	62.5	12	32.6	24.33	≤ 8.805 vs. >8.805	2.66
Hu et al. (29)	China	Retrospective cohort study	ACS after PCI	9,285	75.3	59.9	17.4	43.9	26.2	≤ 8.92 vs. >8.92	2.4
Wang et al. (30)	China	Retrospective cohort study	ACS	2,531	55.9	66.3	36	100	25.9	≤ 8.848 vs. ≥9.383	2.88
Ma et al. (31)	China	Retrospective cohort study	ACS after PCI	776	72.2	61	30	100	26.1	<8.80 vs. ≥9.29	2.4
Zhao et al. (32)	China	Retrospective cohort study	NSTE-ACS after PCI	1,510	73.7	59.7	48	0	25.8	≤ 8.72 vs. >8.72	2.57
Zhao et al. (33)	China	Retrospective cohort study	NSTE-ACS	798	68.3	60.9	36	100	26.7	<9.18 vs. ≥9.18	2.45
Zhang et al. (34)	China	Retrospective cohort study	AMI	1,932	68.5	65.4	26.8	100	25.8	≤ 8.91 vs. >9.54	2.58
Zhang et al. (35)	China	Retrospective cohort study	ACS	1,010	72.8	65	35.6	0	25.5	<8.33 vs. ≥8.33	1.5
Yang et al. (36)	China	Prospective cohort study	CAD after PCI	5,489	79.4	57.2	29	0	25.7	≤ 8.52 vs. ≥8.92	2.49
Zhao et al. (37)	China	Prospective cohort study	NSTE-ACS after PCI	2,107	72	60.02	24	34.2	26.08	≤ 8.87 vs. >8.87	2.52
Gao et al. (38)	China	Prospective cohort study	MINOCA	1,179	73.7	55.7	41.7	15.9	25.47	<8.52 vs. ≥8.99	2.29

*ACS, acute coronary syndrome; AMI, acute myocardial infarction; NSTE, non-ST-segment elevation; PCI, percutaneous coronary intervention; BMI, Body mass index; MINOCA, myocardial infarction with non-obstructive coronary arteries; eGFR, estimated glomerular filtration rate; LDL-C, low-density lipoprotein cholesterol*.

### Primary Outcome

**Figure 2:**
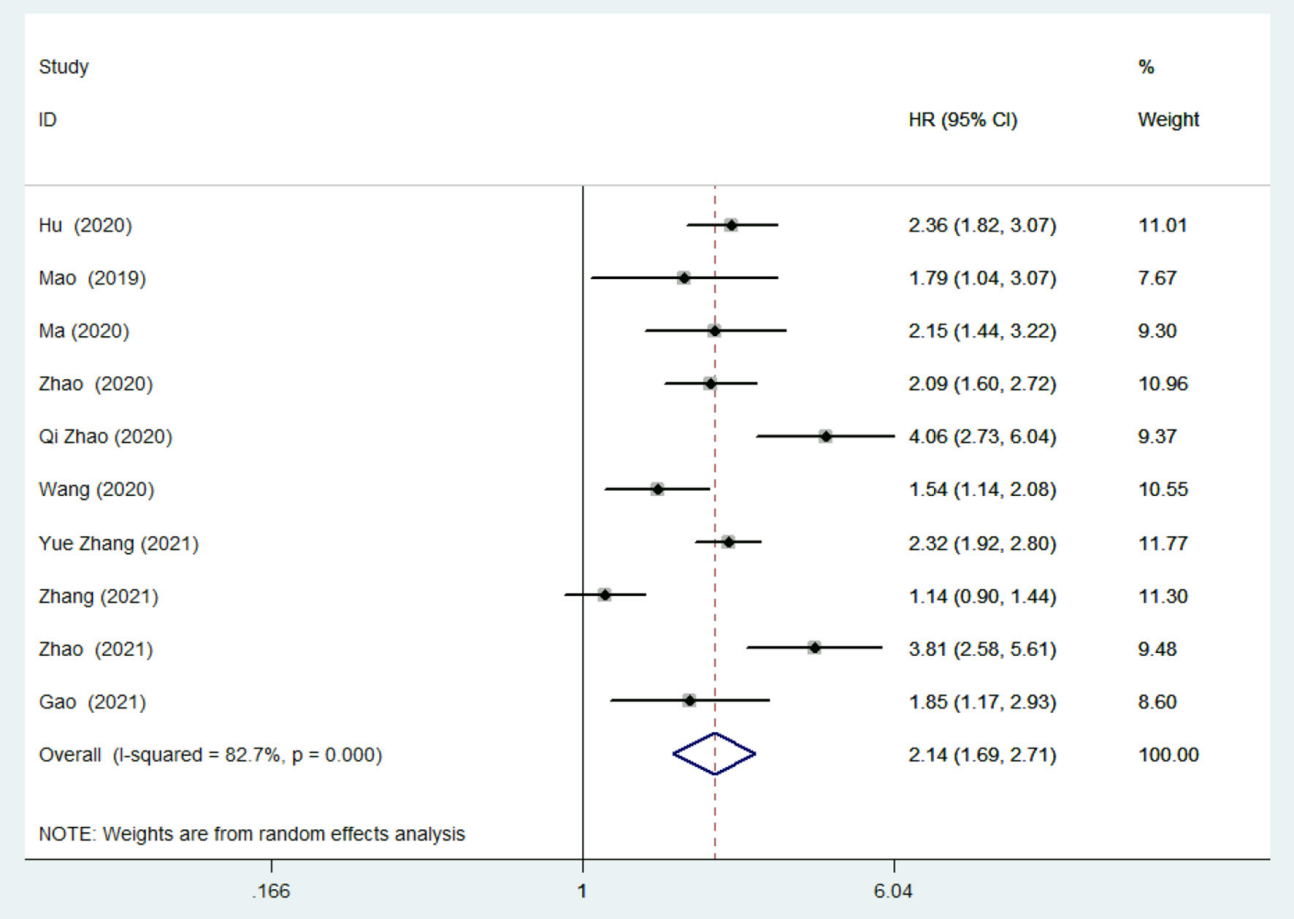
Risk of major adverse cardiac events (MACEs) in the highest Triglyceride-glucose (TyG) index group compared with the lowest TyG index group.

In the meta-analysis of the TyG index evaluated as a categorical variable, 10 studies (28–35, 37, 38) reported the association between the TyG index and the risk of MACEs in patients with CAD. As illustrated in Figure 2, the pooled HR of MACEs comparing the highest TyG index group to the lowest TyG index group was 2.14 (95% CI: 1.69–2.71, *P* < 0.001). Nevertheless, we detected obvious heterogeneity across the 10 studies (*I*^2^ =82.7%, *P* < 0.001). The approximately symmetric funnel plot indicated a low risk of publication bias which was consistent with the Egger's test (*P* = 0.273) (Supplementary File 4). Similarly, in the meta-analysis of TyG index evaluated as a continuous variable, a 1-SD increment in TyG index was associated with 70% higher risk of MACEs (HR: 1.7, 95% CI: 1.37–2.1, *P* < 0.001; heterogeneity: *I*^2^ = 85.6%, *P* < 0.001; Supplementary File 5).

### Secondary Outcomes

Compared with the lowest TyG index group, the highest TyG index group was associated with an increased risk of **MI** (HR: 1.87, 95% CI: 1.46–2.46, *P* < 0.001), **revascularization** (HR: 2.6, 95% CI: 1.76–3.84, *P* < 0.001), and **stroke** (HR: 1.56, 95% CI: 1.06–2.28, *P* = 0.02) in patients with CAD. However, no statistic difference was observed in the analysis of risk of **all-cause death** (HR: 1.33, 95% CI: 0.82-2.17, *P* = 0.245) **or CV death** (HR: 1.87, 95% CI: 0.9–3.88, *P* = 0.094). Evidence of publication bias was detected in the analysis of the risk of all-cause death (Egger's test: *P* = 0.04), but not in the analysis of the risk of MI, revascularization, or stroke by Begg's test or Egger's test (all *P* > 0.05). Table 2 summarizes the HRs for secondary outcomes. Forest plots of secondary outcomes are presented in Supplementary File 6.

**Table 2:** Association between the Triglyceride-glucose (TyG) index and cardiovascular events.

**Outcomes**	**No. of studies**	**HR (95%CI) and *P*-Value**	**Heterogeneity**
**Primary outcome**			
MACEs	10	2.14 (1.69, 2.71), *P* <0.001	82.7%, *P* <0.001
**Secondary outcomes**			
All cause death	5	1.33 (0.82, 2.17), *P* = 0.245	65.4%, *P* = 0.021
CV death	3	1.87 (0.90, 3.88), *P* = 0.094	68.2%, *P* = 0.043
MI	5	1.89 (1.46, 2.46), *P* <0.001	0%, *P* = 0.96
Revascularization	4	2.60 (1.76, 3.84), *P* <0.001	71%, *P* = 0.016
Stroke	4	1.56 (1.06, 2.28), *P* = 0.02	0%, *P* = 0.845

### Sensitivity Analyses, Meta-Regression Analyses, and Subgroup Analyses

In the sensitivity analysis of primary outcome, we found that removing each study did not remarkably reduce the heterogeneity except the study of Zhang et al. (35) which slightly reduced the heterogeneity (*I*^2^ = 66.1%, *P* = 0.003). It should be noted that the study of Zhang et al. (35) differs from others in LDL-C below 1.8 mmol/L. Besides, sensitivity analysis of primary outcome revealed that the removal of any one study did not substantially alter the overall estimation of HR for MACEs. In the sensitivity analysis of secondary outcomes, removing anyone study did not dramatically decrease the heterogeneity among studies, nor did it markedly alter the pooled HR.

Meta-regression analyses of primary outcome did not detect any correlations between HR of MACEs with sample size (*P* = 0.781), age (*P* = 0.245), proportion of male (*P* = 0.654), follow-up duration (*P* = 0.611), and BMI (*P* = 0.074).

In subgroup analyses of the primary outcome, we separated studies according to diabetes status (with DM or without DM), age (<60 or ≥60), BMI (<26 or ≥26), LDL-C (<1.8 or ≥1.8), category of TyG index (median or tertiles), sample size (<2,000 or ≥2,000), follow-up duration (<36 or ≥36) and study design (retrospective or prospective) and focused on the patients after PCI. As demonstrated in Table 3, there was a more pronounced risk of MACEs in populations with higher BMI than in those with lower BMI (*P* = 0.03 for interaction). No significant difference was identified across other subgroups (both *P* > 0.05 for interaction). Notably, the heterogeneity within the subgroup remarkably reduced in patients younger than 60 years old (*I*^2^ = 0%, *P* = 0.624) and moderately reduced in patients after PCI (*I*^2^ = 55.8%, *P* = 0.079) as well as studies in which TyG index was reported as tertiles (*I*^2^ = 44.7%, *P* = 0.143), meaning that age, treatment of PCI and category of TyG index might be the factors contributing to heterogeneity. In the subgroup analyses of secondary outcomes, we stratified studies according to diabetes status. As demonstrated in Table 4, there was no evidence of statistical heterogeneity between diabetic patients and non-diabetic patients in the risk of all-cause death, MI, revascularization, and stroke associated with elevated TyG index (both *P* > 0.05 for interaction).

**Table 3:** Subgroup analyses of the primary outcome.

**Subgroups**	**No. of studies**	**HR (95%CI) and *P-*value**	**Heterogeneity within subgroup**	***P* for interaction**
**Diabetes status**				
With DM	6	2.49 (1.86, 3.34), *P* <0.001	72.9%, *P* = 0.002	0.41
Without DM	4	1.95 (1.20, 3.17), *P < * 0.001	89.2%, *P* <0.001	
**Age**				
<60	3	2.16 (1.82, 2.57), *P* <0.001	0%, *P* = 0.624	0.93
≥60	7	2.17 (1.53, 3.06), *P* <0.001	88.1%, *P* <0.001	
**BMI**				
<26	6	1.74 (1.34, 2.26), *P* <0.001	78.9%, *P* <0.001	0.03
≥26	4	2.94 (2.15, 4.01), *P* <0.001	66.9%, *P* = 0.028	
**Category**				
Median	6	2.30 (1.54, 3.42), *P* <0.001	89.2%, *P* <0.001	0.56
Tertiles	4	1.98 (1.60, 2.45), *P* <0.001	44.7%, *P* = 0.143	
**Sample size**				
<2,000	3	2.05 (1.52, 2.76), *P* <0.001	83.8%, *P* <0.001	0.62
≥2,000	7	2.37 (1.48, 3.77), *P* <0.001	85.0%, *P* = 0.001	
**Follow-Up**				
<36	6	2.11 (1.51, 2.94), *P* <0.001	86.4%, *P* <0.001	0.88
≥36	4	2.20 (1.49, 3.23), *P* <0.001	80.1%, *P* = 0.002	
**Study design**				
Retrospective	7	2.06 (1.57, 2.71), *P* <0.001	85.5%, *P* <0.001	0.63
Prospective	3	2.38 (1.42, 3.99), *P* = 0.001	73.7%, *P* = 0.022	
**LDL-C**				
<1.8	1	1.14 (0.90, 1.44), *P* = 0.28		
≥1.8	9	2.32 (1.92, 2.79), *P* <0.001	66.1%, *P* = 0.003	
**PCI**	4	2.45 (1.94, 3.14), *P* <0.001	55.8%, *P* = 0.079	

**Table 4:** Subgroup analyses of secondary outcomes.

**Subgroups**	**No. of studies**	**HR (95%CI) and *P*-value**	**Heterogeneity within subgroup**	***P* for interaction**
**All-Cause death**				
With DM	3	1.64 (0.95, 2.84), *P* = 0.08	67.8%, *P* = 0.045	0.28
Without DM	2	0.93 (0.53, 1.64), *P* = 0.79	0%, *P* = 0.457	
**MI**				
With DM	3	1.93 (1.41, 2.64), *P* <0.001	0%, *P* = 0.837	0.85
Without DM	2	1.82 (1.13, 2.93), *P* = 0.01	0%, *P* = 0.653	
**Revascularization**				
With DM	2	3.41 (1.68, 6.95), *P =* 0.001	81%, *P* = 0.022	0.31
Without DM	2	2.09 (1.58, 2.76), *P* <0.001	0%, *P* = 0.339	
**Stroke**				
With DM	2	1.65 (1.01, 2.67), *P* = 0.04	0%, *P* = 0.429	0.75
Without DM	2	1.43 (0.77, 2.63), *P* = 0.26	0%, *P* = 0.80	

## Discussion

This meta-analysis shows that the elevated TyG index is associated with increased risk of MACEs, MI, revascularization, and stroke in patients with CAD. A greater risk of MACEs is observed among participants with higher BMI. Nevertheless, the current evidence suggests that the TyG index may not be an indicator of CV mortality or all-cause mortality in patients with CAD.

Consistent with a previous study (39), our findings demonstrated an association between IR the poor cardiovascular outcomes in patients with CAD. In the present study, individuals with a higher TyG index have a 2.14-fold greater risk for MACEs compared with individuals with a lower TyG index. Similarly, in the study of Uetani et al. (39), there was a 1.98-fold higher risk for cardiovascular events among subjects with higher HOMA-IR than those with lower HOMA-IR. Besides, we found a more significant risk of MACEs in patients with higher BMI, which is partly attributed to obesity-linked inflammation and abnormal metabolism (40). Previous works of literature had reported that metabolic disorders, including IR, diabetes, and prediabetes played an important role in the development of CAD (4, 5, 41). A meta-analysis including 129 studies showed that prediabetes was associated with poor prognosis in patients with and without baseline atherosclerotic cardiovascular disease (41). TyG index, a marker of IR, has been reported to be correlated with the development of prediabetes and diabetes mellitus (42, 43). Our subgroup analyses provided supportive evidence that the TyG index was a predictor of MACEs in patients with CAD and diabetes mellitus. Nevertheless, our subgroup analyses did not reveal significant differences across the participants with or without diabetes mellitus (*P* > 0.05 for interaction). Age, the concentration of LDL-C, and treatment of PCI are the suspect factors of heterogeneity. However, we did not detect a significant difference in the subgroup analyses stratified by age. We failed to compare the risk of MACEs between patients with LDL-C below 1.8 mmol/L and patients with above 1.8 mmol/L, and between patients treated with PCI and patients not treated with PCI, due to the limited number of studies. Thus, further studies are needed to elucidate the effect of those suspect factors on the risk of MACEs associated with elevated TyG index in patients with CAD.

In this meta-analysis, five articles (30, 32–35) reported the relationship between the TyG index with all-cause mortality in CAD populations, and our findings showed that the TyG index was not associated with all-cause death in CAD regardless of diabetes status. An analogous result was obtained in the study of Drwila et al. (18) among non-diabetic patients with AMI. In contrast, numerous studies reported the increased risk of all-cause death associated with elevated TyG index. In a retrospective observational study among the general population, compared with the subjects in the lowest TyG index group, those in the highest TyG index group showed a 51% increased risk for all-cause death (44). Similarly, in a study of patients with ischemic stroke, compared with patients in the first quartile of TyG index, those in the fourth quartile of TyG index showed a 25% increase in all-cause mortality (45). Differences in comorbidities of participants and the corresponding drug therapies that improved survival, as well as the publication bias in the analysis of all-cause mortality may interpret this negative finding.

In this meta-analysis, only three studies (28, 34, 35) assessed the relationship between the TyG index with the risk of CV death in CAD populations. Our findings did not show the predictive value of the TyG index for CV death. Similarly, in a study of the general population (44), no significant increase in the occurrence of CV death was observed among subjects with the higher TyG index compared to subjects with the lower TyG index. In contrast, in patients with chronic heart failure and type 2 diabetes, Guo et al. (46) observed that the elevated TyG index was associated with higher CV mortality. These conflicting results might be partly attributed to the limited number of studies enrolled in our analysis and the heterogeneity of study populations. In the study by Liu et al. (44), a general population was analyzed. While in the study by Guo et al. (46), patients with chronic heart failure and type 2 diabetes were analyzed. Health status may affect the relationship of the TyG index with the risk of CV death. Moreover, for patients with CAD, the strengthened control of cardiovascular risk factors reduced the risk of CV death. Thus, changes in the TyG index over time should be examined and monitored.

In the present study, compared with the lowest TyG index group, patients in the highest TyG index group have a 1.89-fold risk and 2.6-fold risk for MI and revascularization, respectively. Similarly, in a prospective cohort study with 98,849 subjects, there was a 2-fold higher risk for MI in participants with a higher TyG index (47). Accumulating evidence indicated that the elevated TyG index was linked to accelerated atherosclerosis. The study of Won et al. (11) found that patients with elevated TyG index had a more remarkable increase in coronary plaque volume. The study of Lee et al. (48) demonstrated that there was a >3-fold increase in the incidence of coronary artery stenosis in subjects with elevated TyG index. Several cross-sectional observational studies among the Chinese and Korean populations illustrated that the TyG index was linked to arterial stiffness (49, 50). Moreover, an elevated TyG index was positively correlated with in-stent restenosis in patients after drug-eluting stent (51). These data supported that a high TyG index was closely related to accelerated progression of CAD, cumulatively leading to a high risk of revascularization.

As for the risk of stroke associated with elevated TyG index, an earlier meta-analysis showed that a higher TyG index was associated with a 26% increased risk of stroke among participants without atherosclerotic cardiovascular diseases at baseline (52). Similarly, in a cross-sectional study with a general population, the incidence of ischemic stroke increased by 22.8% for each additional SD in the TyG index (53). Our results are consistent with the earlier findings that elevated TyG index is related to the increased risk of stroke in patients with CAD. Nevertheless, this association was not observed in non-diabetic patients in our subgroup analyses. The difference between the two subgroups (with DM or without DM) was not statistically significant (*P* = 0.75 for interaction). To confirm the predictive role of the TyG index in non-diabetic patients with CAD, further studies are needed.

It has been reported that IR, prediabetes, and diabetes may mediate the development and progression of heart failure (54, 55). A meta-analysis comprising 28,643 participants showed that prediabetes was associated with worse outcomes in patients with heart failure (55). TyG index, a biomarker of IR, prediabetes, and diabetes, may be linked with the occurrence of heart failure. Indeed, a recent study demonstrated that the TyG index was positively associated with myocardial fibrosis as well as the risk of all-cause mortality and heart failure hospitalization in patients with heart failure (56). However, in this meta-analysis, the relationship between TyG index and heart failure in patients with CAD was not evaluated since only two studies (28, 38) reported the risk of heart failure. In the study by Mao et al. (28), no difference was observed in the occurrence of heart failure between the high TyG index group and the low TyG index group. The consistent result was shown in the study by Gao et al. (38) that the incidence of hospitalization for heart failure did not increase in parallel with the TyG index tertiles (*P* = 0.08). The predictive role of the TyG index for heart failure in patients with CAD needs further assessment.

There are several limitations in the present study. First, all the participates included in our study were from China. The prognostic role of the TyG index for CAD in other countries remains unclear. Second, there were a small number of studies assessing the relationship of the TyG index with the risk of CV death, revascularization, and stroke in individuals with CAD. Additionally, the predictive capacity of the TyG index for adverse events is not separately analyzed in different types of CAD due to a limited number of studies. A study on the prognostic significance of the TyG index in chronic coronary syndrome is lacking. Finally, our analysis only focused on the prognostic value of the baseline TyG index in CAD. The effect of longitudinal changes in the TyG index on the risk of MACEs in patients with CAD remains uncertain. Thus, one should be cautious to interpret the results of our study. More large-scale prospective studies should be conducted to validate the prognostic significance of the TyG index for adverse cardiovascular outcomes in patients with CAD.

## Conclusion

In this meta-analysis, the elevated TyG index was found to be closely associated with increased occurrence of MACEs, MI, revascularization, and stroke in patients with CAD, and a stronger risk of MACEs was observed in patients with higher BMI. These data provided a rationale to consider the elevated TyG index as a valuable predictor of cardiovascular events in CAD. Measuring the TyG index could contribute to risk identification and proper management in patients with CAD.

## Data Availability Statement

The original contributions presented in the study are included in the article/Supplementary Material, further inquiries can be directed to the corresponding author/s.

## Author Contributions

J-WL, W-HD, and D-ZS conceived the idea of this meta-analysis. J-WL developed a protocol with the assistance of W-HD. J-WL and Y-QY performed the data analyses independently. J-WL and LS participated in the data interpretation and drafting of the manuscript. D-ZS and W-HD critically reviewed the manuscript. All the authors had browsed and approved the final version of the manuscript.

## Funding

This study is supported by Innovation Team and Talents Cultivation Program of National Administration of Traditional Chinese Medicine (No: ZYYCXTD-C-202007).

## Conflict of Interest

The authors declare that the research was conducted in the absence of any commercial or financial relationships that could be construed as a potential conflict of interest.

## Publisher's Note

All claims expressed in this article are solely those of the authors and do not necessarily represent those of their affiliated organizations, or those of the publisher, the editors and the reviewers. Any product that may be evaluated in this article, or claim that may be made by its manufacturer, is not guaranteed or endorsed by the publisher.
