# Supplementary material for: Prognostic Significance of Triglyceride-Glucose Index for Adverse Cardiovascular Events in Patients With Coronary Artery Disease: A Systematic Review and Meta-Analysis
Source: Front Cardiovasc Med. 2021 Dec 2;8:774781. doi: 10.3389/fcvm.2021.774781 (PMC8674619; doi:10.3389/fcvm.2021.774781)

Supplementary File S1. Search strategy for PubMed

#1	"triglyceride glucose index"[Title/Abstract] OR "triglyceride glucose index"[Title/Abstract] OR "tyg index"[Title/Abstract]
#2	"coronary disease"[MeSH Terms] OR "coronary diseases"[Title/Abstract] OR "disease coronary"[Title/Abstract] OR "diseases coronary"[Title/Abstract] OR "coronary heart disease"[Title/Abstract] OR "coronary heart diseases"[Title/Abstract] OR "disease coronary heart"[Title/Abstract] OR "diseases coronary heart"[Title/Abstract] OR "heart disease coronary"[Title/Abstract] OR "heart diseases coronary"[Title/Abstract]
#3	"coronary artery disease"[MeSH Terms] OR "artery disease coronary"[Title/Abstract] OR "artery diseases coronary"[Title/Abstract] OR "coronary artery diseases"[Title/Abstract] OR "left main coronary artery disease"[Title/Abstract] OR "left main disease"[Title/Abstract] OR "left main diseases"[Title/Abstract] OR "left main coronary disease"[Title/Abstract] OR "coronary arteriosclerosis"[Title/Abstract] OR (("Arteriosclerosis"[MeSH Terms] OR "Arteriosclerosis"[All Fields] OR "Arterioscleroses"[All Fields]) AND "Coronary"[Title/Abstract]) OR (("coronaries"[All Fields] OR "heart"[MeSH Terms] OR "heart"[All Fields] OR "Coronary"[All Fields]) AND "Arterioscleroses"[Title/Abstract]) OR "atherosclerosis coronary"[Title/Abstract] OR (("Atherosclerosis"[MeSH Terms] OR "Atherosclerosis"[All Fields] OR "Atheroscleroses"[All Fields]) AND "Coronary"[Title/Abstract]) OR (("coronaries"[All Fields] OR "heart"[MeSH Terms] OR "heart"[All Fields] OR "Coronary"[All Fields]) AND "Atheroscleroses"[Title/Abstract]) OR "coronary atherosclerosis"[Title/Abstract] OR "arteriosclerosis coronary"[Title/Abstract]
#4	"acute coronary syndrome"[MeSH Terms] OR "acute coronary syndromes"[Title/Abstract] OR "coronary syndrome acute"[Title/Abstract] OR "coronary syndromes acute"[Title/Abstract] OR "syndrome acute coronary"[Title/Abstract] OR "syndromes acute coronary"[Title/Abstract] OR "ACS"[Title/Abstract] OR "myocardial infarction"[MeSH Terms] OR "MI"[Title/Abstract] OR "infarction myocardial"[Title/Abstract] OR "infarctions myocardial"[Title/Abstract] OR "myocardial infarctions"[Title/Abstract] OR "cardiovascular stroke"[Title/Abstract] OR (("cardiovascular system"[MeSH Terms] OR ("Cardiovascular"[All Fields] AND "system"[All Fields]) OR "cardiovascular system"[All Fields] OR "Cardiovascular"[All Fields] OR "cardiovasculars"[All Fields]) AND "Strokes"[Title/Abstract]) OR "stroke cardiovascular"[Title/Abstract] OR "strokes cardiovascular"[Title/Abstract] OR "myocardial infarct"[Title/Abstract] OR "infarcts myocardial"[Title/Abstract] OR "myocardial infarcts"[Title/Abstract] OR "heart attack"[Title/Abstract] OR "heart attacks"[Title/Abstract] OR "stemi st segment elevation myocardial infarction"[Title/Abstract] OR "non st elevation acute coronary syndrome"[Title/Abstract] OR "non st segment elevation myocardial infarction"[Title/Abstract] OR "NSTEMI"[Title/Abstract] OR "NSTE-ACS"[Title/Abstract] OR "angina, stable"[MeSH Terms] OR (("angina pectoris"[MeSH Terms] OR ("Angina"[All Fields] AND "Pectoris"[All Fields]) OR "angina pectoris"[All Fields] OR "Angina"[All Fields] OR "Anginas"[All Fields]) AND "Stable"[Title/Abstract]) OR "stable angina"[Title/Abstract] OR (("Stable"[All Fields] OR "stabled"[All Fields] OR "stables"[All Fields] OR "stabling"[All Fields]) AND "Anginas"[Title/Abstract]) OR "chronic stable angina"[Title/Abstract] OR "angina chronic stable"[Title/Abstract] OR (("angina pectoris"[MeSH Terms] OR ("Angina"[All Fields] AND "Pectoris"[All Fields]) OR "angina pectoris"[All Fields] OR "Angina"[All Fields] OR "Anginas"[All Fields]) AND "chronic stable"[Title/Abstract]) OR ((("Chronic"[All Fields] OR "chronical"[All Fields] OR "chronically"[All Fields] OR "chronicities"[All Fields] OR "chronicity"[All Fields] OR "chronicization"[All Fields] OR "chronics"[All Fields]) AND ("Stable"[All Fields] OR "stabled"[All Fields] OR "stables"[All Fields] OR "stabling"[All Fields])) AND "Anginas"[Title/Abstract]) OR "stable angina chronic"[Title/Abstract] OR (("angina, stable"[MeSH Terms] OR ("Angina"[All Fields] AND "Stable"[All Fields]) OR "stable angina"[All Fields] OR ("Stable"[All Fields] AND "Anginas"[All Fields])) AND "Chronic"[Title/Abstract]) OR "angina pectoris stable"[Title/Abstract] OR ((("angina pectoris"[MeSH Terms] OR ("Angina"[All Fields] AND "Pectoris"[All Fields]) OR "angina pectoris"[All Fields] OR "Angina"[All Fields] OR "Anginas"[All Fields]) AND "Pectori"[All Fields]) AND "Stable"[Title/Abstract]) OR "pectoris stable angina"[Title/Abstract] OR (("angina, stable"[MeSH Terms] OR ("Angina"[All Fields] AND "Stable"[All Fields]) OR "stable angina"[All Fields] OR ("Stable"[All Fields] AND "Angina"[All Fields])) AND "Pectori"[Title/Abstract]) OR "stable angina pectoris"[Title/Abstract] OR "angina, unstable"[MeSH Terms] OR (("angina pectoris"[MeSH Terms] OR ("Angina"[All Fields] AND "Pectoris"[All Fields]) OR "angina pectoris"[All Fields] OR "Angina"[All Fields] OR "Anginas"[All Fields]) AND "Unstable"[Title/Abstract]) OR "unstable anginas"[Title/Abstract] OR "angina pectoris unstable"[Title/Abstract] OR ((("angina pectoris"[MeSH Terms] OR ("Angina"[All Fields] AND "Pectoris"[All Fields]) OR "angina pectoris"[All Fields] OR "Angina"[All Fields] OR "Anginas"[All Fields]) AND "Pectori"[All Fields]) AND "Unstable"[Title/Abstract]) OR (("angina, unstable"[MeSH Terms] OR ("Angina"[All Fields] AND "Unstable"[All Fields]) OR "unstable angina"[All Fields] OR ("Unstable"[All Fields] AND "Angina"[All Fields])) AND "Pectori"[Title/Abstract]) OR "unstable angina pectoris"[Title/Abstract] OR "unstable angina"[Title/Abstract] OR "angina at rest"[Title/Abstract] OR "angina preinfarction"[Title/Abstract] OR (("angina pectoris"[MeSH Terms] OR ("Angina"[All

	Fields] AND "Pectoris"[All Fields]) OR "angina pectoris"[All Fields] OR "Angina"[All Fields] OR "Anginas"[All Fields]) AND "Preinfarction"[Title/Abstract]) OR "preinfarction angina"[Title/Abstract] OR (("preinfarct"[All Fields] OR "Preinfarction"[All Fields]) AND "Anginas"[Title/Abstract]) OR (("myocardially"[All Fields] OR "myocardium"[MeSH Terms] OR "myocardium"[All Fields] OR "Myocardial"[All Fields]) AND "preinfarction syndrome"[Title/Abstract]) OR (("myocardially"[All Fields] OR "myocardium"[MeSH Terms] OR "myocardium"[All Fields] OR "Myocardial"[All Fields]) AND "preinfarction syndromes"[Title/Abstract]) OR (("preinfarct"[All Fields] OR "Preinfarction"[All Fields]) AND "syndrome myocardial"[Title/Abstract]) OR (("preinfarct"[All Fields] OR "Preinfarction"[All Fields]) AND "syndromes myocardial"[Title/Abstract]) OR ((("syndrom"[All Fields] OR "syndromal"[All Fields] OR "syndromally"[All Fields] OR "Syndrome"[MeSH Terms] OR "Syndrome"[All Fields] OR "Syndromes"[All Fields] OR "syndrome s"[All Fields] OR "syndromic"[All Fields] OR "syndroms"[All Fields]) AND ("myocardially"[All Fields] OR "myocardium"[MeSH Terms] OR "myocardium"[All Fields] OR "Myocardial"[All Fields])) AND "Preinfarction"[Title/Abstract]) OR ((("syndrom"[All Fields] OR "syndromal"[All Fields] OR "syndromally"[All Fields] OR "Syndrome"[MeSH Terms] OR "Syndrome"[All Fields] OR "Syndromes"[All Fields] OR "syndrome s"[All Fields] OR "syndromic"[All Fields] OR "syndroms"[All Fields]) AND ("myocardially"[All Fields] OR "myocardium"[MeSH Terms] OR "myocardium"[All Fields] OR "Myocardial"[All Fields])) AND "Preinfarction"[Title/Abstract])
#5	#2 OR #3 OR #4
#6	#5 AND #6

Supplementary File S2. Methodological quality assessment of 12 studies

Study	Selection				Comparability		Outcome			Score
	exposed cohort	non-exposed cohort	ascertainment of exposure	demonstration that outcome of interest was not present at start of study	important confounder adjusted	other confounders adjusted	assessment of outcome	duration of follow-up	adequacy of follow up	
Jin 2018 (27)	1	1	1	1	1	0	1	1	1	8
Mao 2019 (28)	1	1	1	1	1	1	1	1	1	9
Hu 2020 (29)	1	1	1	1	1	0	1	1	1	8
Wang 2020 (30)	1	1	1	1	1	0	1	1	1	8
Ma 2020 (31)	1	1	1	1	1	1	1	1	1	9
Zhao 2020 (32)	1	1	1	1	1	1	1	1	1	9
Qi Zhao 2020 (33)	1	1	1	1	1	1	1	1	1	9
Yue Zhang 2021 (34)	1	1	1	1	1	1	1	1	1	9
Zhang 2021 (35)	1	1	1	1	1	1	1	1	1	9
Yang 2021 (36)	1	1	1	1	1	1	1	1	1	9
Zhao 2021 (37)	1	1	1	1	1	1	1	1	1	9
Gao 2021 (38)	1	1	1	1	1	0	1	1	1	8

Supplementary File S3. The definition of MACEs and adjusted covariates in individual enrolled study

Study	MACEs	Adjusted covariates
Jin 2018 (27)	all-cause death, non-fatal MI, stroke and post-discharge revascularization (PCI or CABG)	BMI, LVEF, hypertension, DM, uric acid, smoke, hsCRP, HDL-C and LDL-C
Mao 2019 (28)	cardiac death, non-fatal MI, target vessel revascularization, heart failure, and non-fatal stroke	age, gender, metabolic syndrome, LDL-C, HDL-C, SYNTAX score, CRP, basal insulin, sulfonylurea, metformin, α-glucosidase inhibitor, ACEI/ARB, beta-blocker, and PCI/CABG
Hu 2020 (29)	death, non-fatal MI, non-fatal stroke or unplanned repeat revascularization	age, sex, BMI, current smoker, hypertension, previous MI, previous stroke, previous PCI, previous CABG, and ACS status
Wang 2020 (30)	all-cause death, non-fatal MI and non-fatal stroke	age, male, smoker, previous MI, previous CABG, BMI, acute MI, LVEF, left main disease, multi-vessel disease, HbA1c, hs-CRP, statin, insulin
Ma 2020 (31)	all-cause death, non-fatal stroke, non-fatal MI, or unplanned repeat revascularization	age, BMI, DBP, HDL-C, glycosylated haemoglobin (continuous), sex, current smoking, daily drinking, presence of PAD, CKD, cardiac failure, previous MI, past PCI, use of insulin and/or oral antidiabetic agents at discharge, CAD severity, presence of lesions > 20 mm long, use of drug-coated balloon, and complete revascularization
Zhao 2020 (32)	all-cause death, non-fatal MI, non-fatal ischemic stroke, and ischemia-driven revascularization	age, gender, BMI, smoking history, hypertension, dyslipidemia, previous history of MI, PCI, stroke, PAD, TC, HDL-C, eGFR, HbA1, LVEF, left main artery disease, three-vessel disease, chronic total occlusion, diffuse lesion, in-stent restenosis, SYNTAX score, treatment of LM, left circumflex artery, right coronary artery, drug-eluting stent implantation, drug-coated balloon application, complete revascularization, number of stents, dual antiplatelet therapy at admission, statins at admission, and ACEI/ARB at discharge
Qi Zhao 2020 (33)	all-cause death, non-fatal MI and ischemia-driven revascularization	age, sex, BMI, SBP, DBP, smoking, drinking, duration of diabetes, dyslipidemia, prior MI, PCI, stroke and PVD, diagnosis (NSTEMI), TC, HDL-C, eGFR, HbA1c, LVEF, SYNTAX score, LM treatment, drug-coated balloon use, complete revascularization and number of stents
Yue Zhang 2021 (34)	all-cause death, non-fatal MI, non-fatal stroke, cardiac rehospitalization (admission because of angina or heart failure), and revascularization	age, BMI, history of stroke and PCI, antiplatelet agent used before admission, WBC, hemoglobin, albumin, eGFR, LVEF, angiography findings (LM/three-disease and proximal LAD), in-hospital treatment (PCI/CABG, antiplatelet agent, ACEI/ARB, beta-blocker, statins) and hypoglycemic agents
Zhang 2021 (35)	all-cause deaths, CV death, non-fatal MI, non-fatal stroke, revascularization, cardiac rehospitalization	age, gender, BMI, BP, heart rate, smoke, hypertension, old MI, MestS, ACEI/ARB, antiplatelet agent, CCB, diuretics, beta-blocker, statins, white blood cell, neutrophil ratio, hemoglobin, HbA1c, creatinine, eGFR, TC, TG, HDL-C, LDL-C, LVEF
Yang 2021 (36)	all-cause death, non-fatal MI, non-fatal stroke, and target vessel revascularization	age, gender, dyslipidemia, previous history of stroke, MI, PCI, CABG, Multivessel disease, chronic total occlusion disease, in-stent restenosis, SYNTAX score, numbers of stent, LVEF, FBG, HbA1c and hs-CRP

Zhao 2021 (37)	cardiac death, non-fatal myocardial infarction, and non-fatal ischemic stroke	smoking history, hypertension, T2DM, previous MI, previous PCI, previous stroke, clinical diagnosis, TC, hs-CRP, eGFR, HbA1c, LVEF, ACEI/ARB at discharge, oral antidiabetic agents at discharge, insulin at discharge, left main artery disease, three-vessel disease, chronic total occlusion, SYNTAX score, complete revascularization, and number of stents
Gao 2021 (38)	all-cause death, non-fatal MI, revascularization, non-fatal stroke, and hospitalization for unstable angina or heart failure	age, sex, MI type, hypertension, diabetes and dyslipidemia

BMI, body mass index; LVEF, left ventricular ejection fraction; DM, diabetes mellitus; hsCRP, high sensitive C-reactive protein; LDL-C, low density lipoprotein cholesterol; HDL-C, high density lipoprotein cholesterol; PCI, percutaneous coronary intervention; CABG, Coronary artery bypass grafting; ACS, acute coronary syndrome; MI, myocardial infarction; HbA1c, glycosylated hemoglobin A1c; PAD, peripheral artery disease, CKD, chronic kidney disease; CAD, coronary artery disease; DBP, diastolic blood pressure; SBP, systolic blood pressure; eGFR, estimated glomerular filtration rate; ACEI, angiotensin-converting enzyme inhibitor; ARB, angiotensin receptor blocker; T2DM, type 2 diabetes mellitus; FBG, fasting blood glucose; TG, triglyceride; SYNTAX, synergy between PCI with taxus and cardiac surgery

Supplementary File S4. Funnel plot for primary outcome

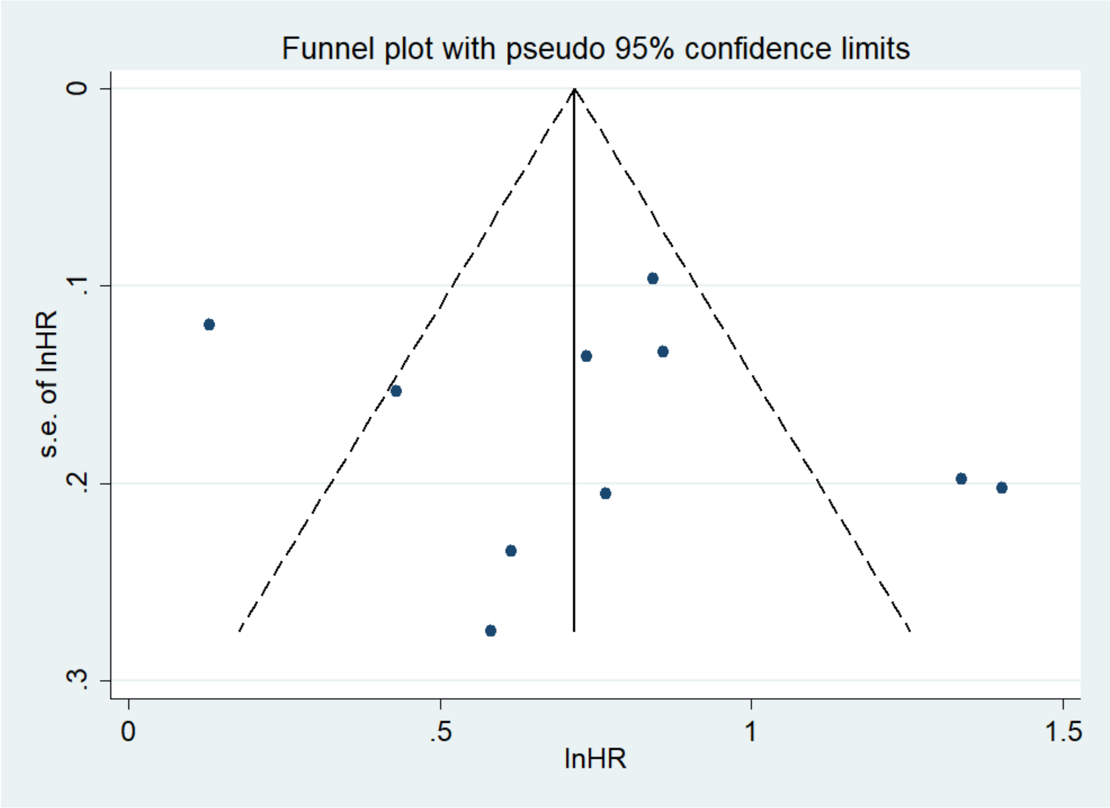

Supplementary File S5. Forest plot of primary outcome. TyG index was evaluated as a continuous variable

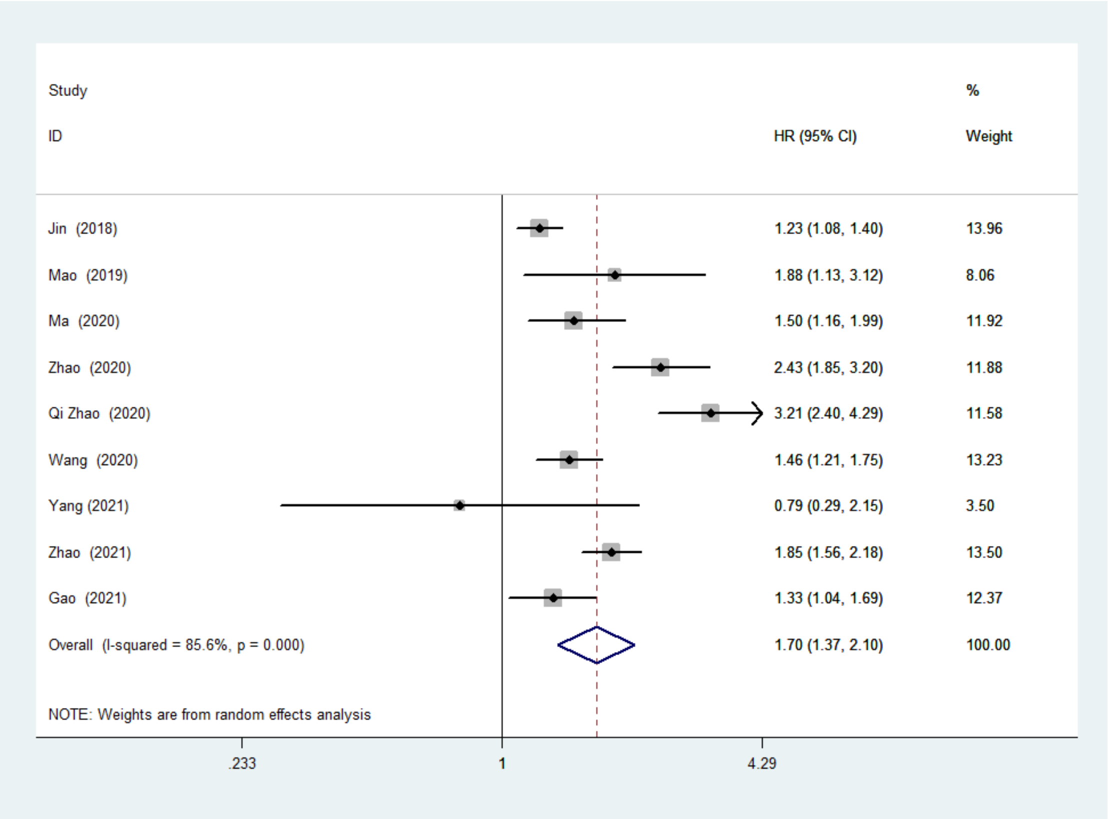

Supplementary File S6. Forest plots of secondary outcomes. (a) all-cause death, (b) CV death, (c) MI, (d) revascularization, (e) stroke

(a) all-cause death

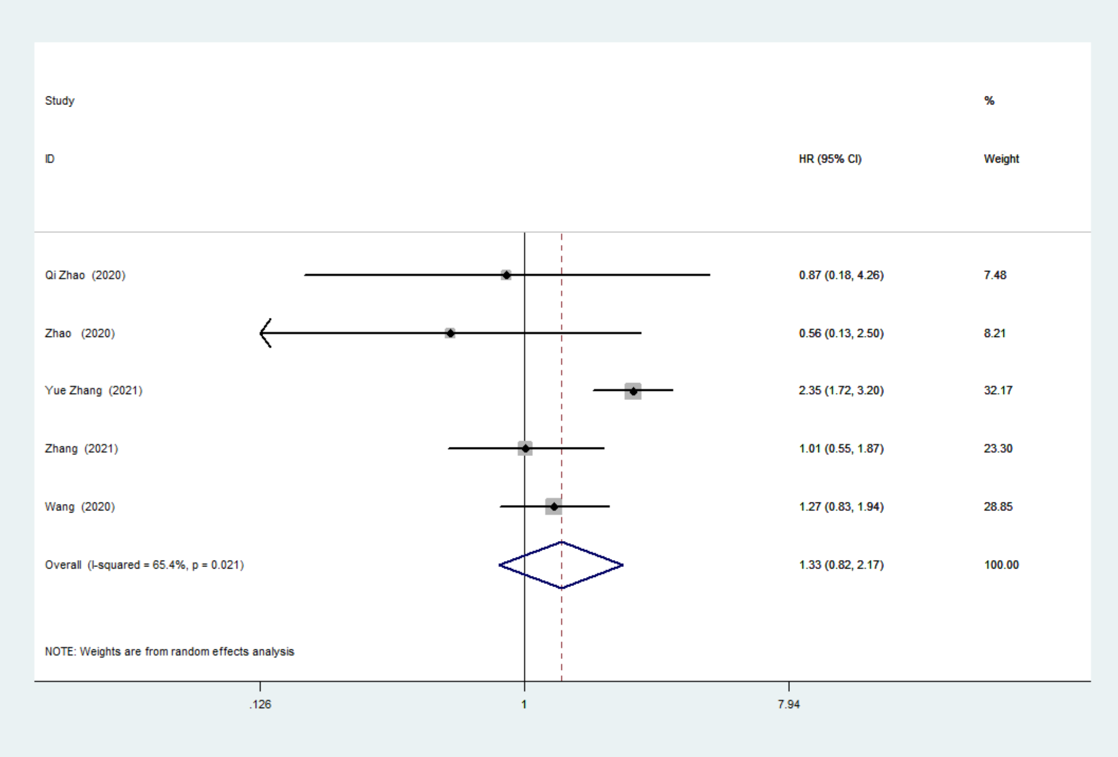

(b) CV death

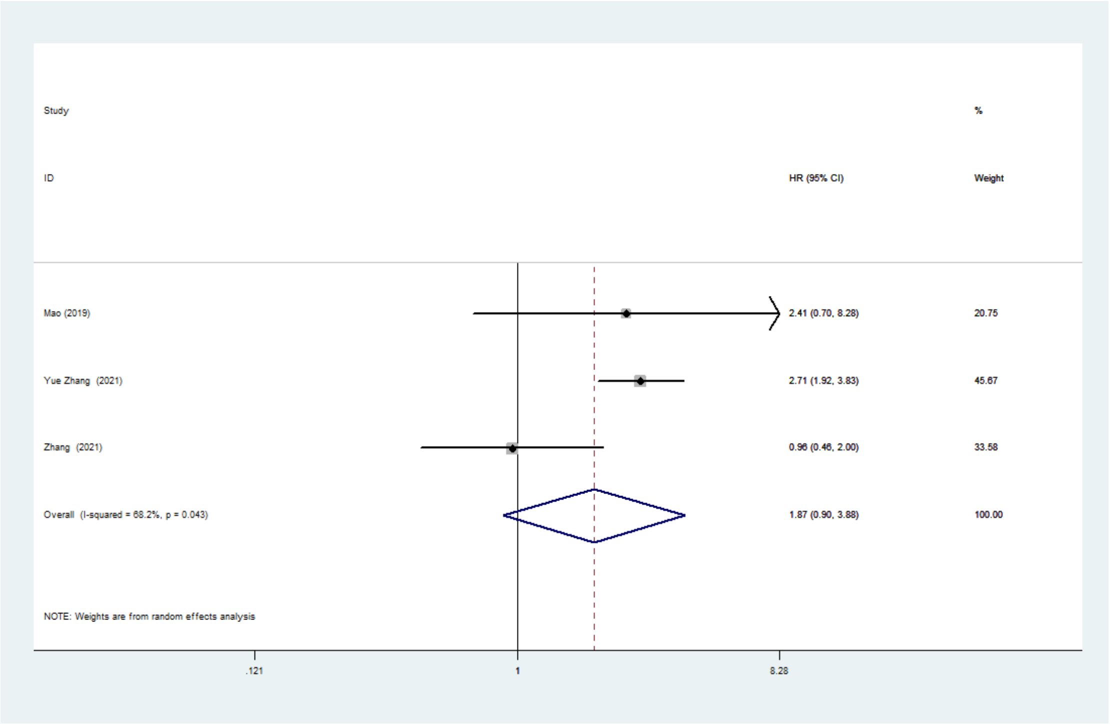

(c) MI

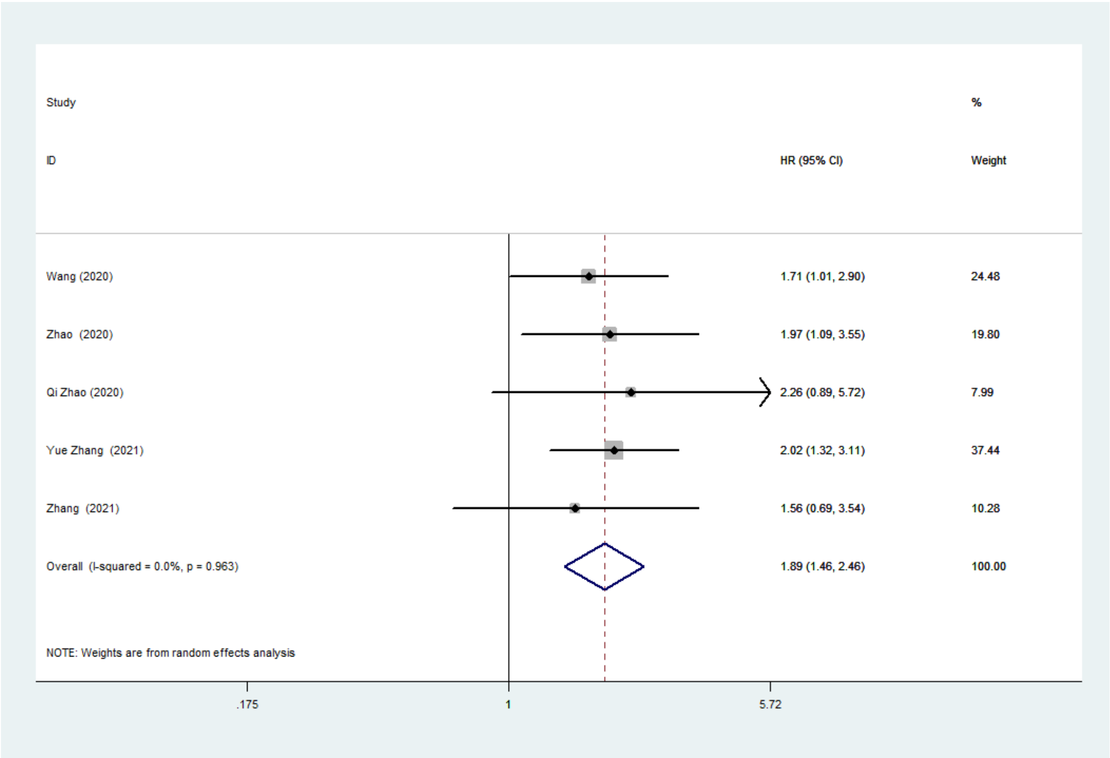

(d) revascularization

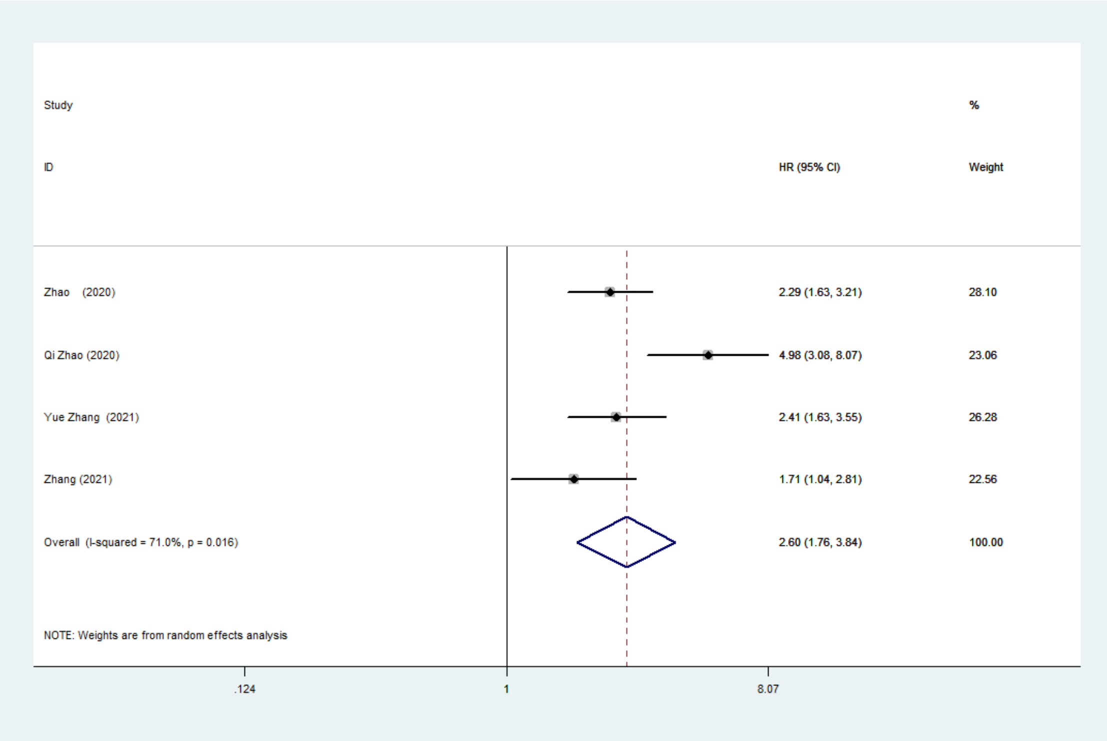

(e) stroke

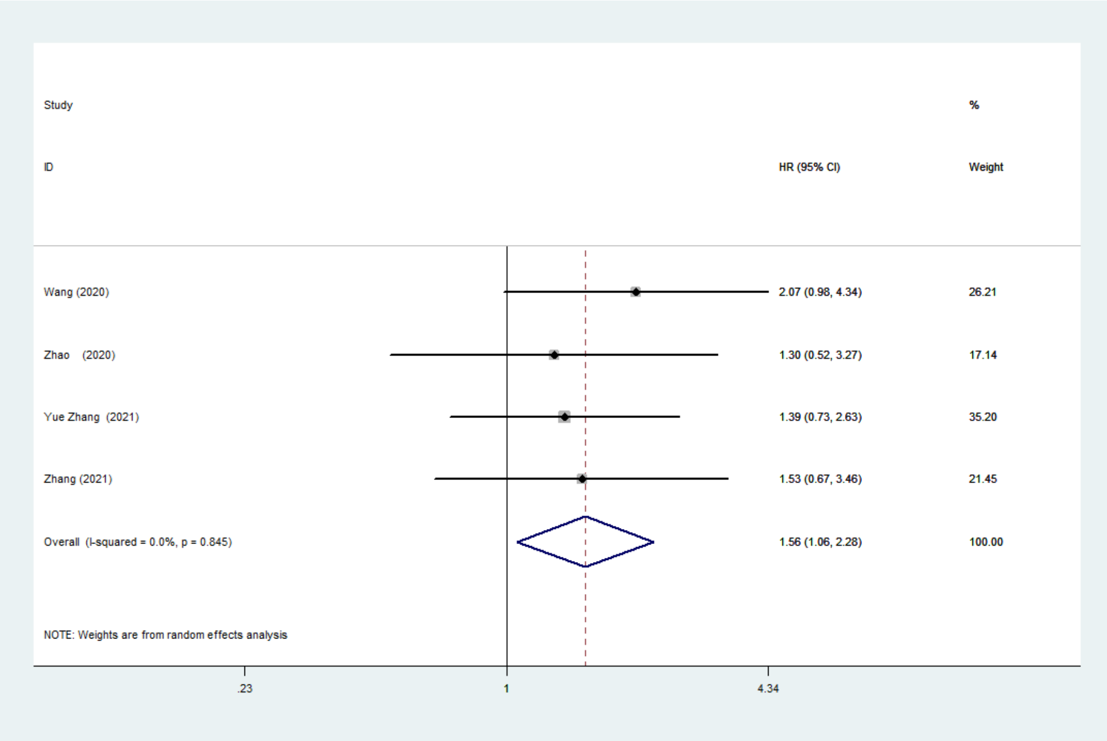

Supplement: Supplementary file 1 [file Data_Sheet_1.PDF]
